# Comparative Studies on Thickeners as Hydraulic Fracturing Fluids: Suspension versus Powder

**DOI:** 10.3390/gels8110722

**Published:** 2022-11-08

**Authors:** Shenglong Shi, Jinsheng Sun, Kaihe Lv, Jingping Liu, Yingrui Bai, Jintang Wang, Xianbin Huang, Jiafeng Jin, Jian Li

**Affiliations:** 1 Department of Petroleum Engineering, China University of Petroleum (East China), Qingdao 266580, China; 2 CNPC Engineering Technology R&D Company Limited, Beijing 102206, China

**Keywords:** hydraulic fracturing, suspension, nano-silica, powder thickener, network structure

## Abstract

To overcome the problems of long dissolution time and high investment in surface facilities of powder thickeners in hydraulic fracturing, a novel suspension of a thickener as a fracturing fluid was prepared using powder polyacrylamide, nano-silica, and polyethylene glycol by high-speed mixing. The suspension and powder were compared in terms of properties of solubility, rheological behavior, sand carrying, drag reduction, and gel breaking. The results showed that the suspension could be quickly diluted in brine within 5 min, whereas the dissolution time of powder was 120 min. The suspension exhibited better performance in salt resistance, temperature resistance, shear resistance, viscoelasticity, sand carrying, and drag reduction than powder. The powder solution was broken more easily and had a lower viscosity than suspension diluent. These improvements in properties of the suspension were due to the dispersion of nano-silica in the polymer matrix; the mobility of thickener chains was inhibited by the steric hindrance of the nano-silica. Nano-silica particles acted as crosslinkers by attaching thickener chains, which strengthened the network structure of the thickener solution. The presence of hydrogen bonds between the thickener matrix and the nano-silica restricted the local movement of thickener chains, leading to a stronger spatial network. Therefore, this novel suspension showed good potential for fracturing applications.

## 1. Introduction

With the continuous exploration and development of conventional oil and gas resources, the quantity of these resources is gradually reduced. Therefore, unconventional oil and gas reservoirs such as shale and tight reservoirs, coal gas, and methane hydrate have been receiving more and more attention [1,2]. Fracturing technology is a key step in oil exploitation and formation stimulation of these reservoirs [3,4]. During hydraulic fracturing, large quantities of fracturing fluid are pumped into the wellbore to maintain formation pressure. When the bottom hole pressure exceeds the formation fracture pressure, the formation is damaged to form an artificial fracture, and fracturing fluid is injected into the fracture. The proppant particles are carried by fracturing fluid and transported into the artificial fractures. After hydraulic fracturing, the fracturing fluid is broken into a low-viscosity liquid by gel breakers and flows back to the ground, minimizing fluid damage to the formation and proppant pack fracture. Meanwhile, proppants are held in the crack and will not flow back to the surface. After pumping, the formation pressure decreases and the fracture walls become close to each other. However, proppants keep the fractures open, allowing for the release of more hydrocarbons and higher oil well productivity [5,6,7,8].

The performance of fracturing fluids determines the success or failure of fracturing operations [9,10]. Commonly used fracturing fluids include water-based, oil-based, acid-based, and foam-based fracturing fluids. Water-based fracturing fluid is the most widely used fracturing fluid. Water-based fracturing fluid plays a leading role in hydraulic fracturing due to its environmental friendliness and safety. The optimum water-based fracturing fluids have the following characteristics: high temperature stability, shear resistance, good sand-carrying capacity, low friction, low fluid loss, low damage, and ability to break and clean up quickly once the treatment is completed [11,12]. As the vital component of water-based fracturing fluid, thickeners can reduce pumping pressure and improve sand-carrying capacity [13,14]. The performance of the thickener is especially important in volumetric fracturing technology, which is indirectly related to the success of oil and gas well fracturing [15].

Thickeners are generally high-molecular-weight polypropylene or its copolymer. The common polymer thickeners are divided into emulsions and powders on the basis of morphology [16]. Powder thickeners are the most effective and they are easy to store and suitable for long-distance transportation, but the swelling and dissolving time of powders is long and central polymer-preparation equipment is generally required, which brings certain difficulties for onsite operation [17]. Therefore, emulsion products are extensively used in thickener preparation, including water-in-oil (W/O) emulsions and water-in-water (W/W) emulsions. W/W emulsions was prepared by aqueous dispersion polymerization, using water instead of oil as a solvent. During the reaction process, the reacting monomers and polymer products are homogeneously suspended by adding small amounts of dispersants and media modifiers at the beginning. The final product is an aqueous solution containing a homogeneous and stable dispersion of polymer particles with a particle size of 10 μm or less and a solid content of 20–40%. Emulsions can be easily dispersed and dissolved in water without needing specific equipment [18]. However, the synthesis of W/O polyacrylamide requires large amounts of the oil phase, which poses a threat to the environment after flowing back to the surface [19,20]. W/W emulsions swell due to the existence of the water phase, which decreases the polymer stability and is detrimental to long-term storage and transportation [21,22].

In recent years, a novel suspension of a thickener as a fracturing fluid with the characteristics of rapid dissolution, environmental friendliness, and suitability for long-term storage and transportation has been reported [23,24,25]. The suspension mainly uses organic alcohol or white oil as the dispersion medium, with the addition of an emulsifier, anti-sedimentation agent, and other additives to form a stable suspension base liquid, and then polyacrylamide powders are added to prepare a suspension system by high-speed mixing, which is used for continuous mixing. Wang et al. [24] developed a low-damage hydrophilic suspension friction reducer system dissolvable in polyethylene glycol 200; a mixture of polyamide wax and modified bentonite was used as an anti-sedimentation agent, the suspension friction reducer system could stand for 30 days without any sedimentation, the viscosity of the suspension system was maintained at 177 mPa·s, the suspension system could be dispersed into water uniformly, the viscosity of the slick water was adjustable, and the drag reduction rate was larger than 70%. Liu et al. [25] optimized the polymer powder size, dispersant, anti-sedimentation agent, and hydrocarbon continuous phase to prepare a suspension system. The proportion of powder accounted for 45% of the suspension system, which could be dissolved in high-salinity brine to prepare a fracturing fluid; the dissolution time was less than 30 s. The viscosity of the suspension system can be adjusted in real time by changing the concentration, and the slick water system can be quickly transformed into a gel system. Therefore, the suspension has the potential to replace emulsion thickeners.

Polymer thickeners are affected by thermal degradation and precipitation under high-temperature and high-salinity conditions [26,27]. Additionally, they usually suffer from shear degradation under turbulent-flow conditions [28]. To overcome these shortcomings, researchers can enhance the temperature, salinity, and shear resistance of the polymer by introducing monomers with a ring structure, betaine, strong electrolyte group, and hydrophobic structure into the backbone of acrylamide [29,30,31]. Mao et al. [29] reported a quaternary thickener P (acrylamide/acrylic acid/2-acrylamide-2-methylpropanesulfonic acid/hydrophobic monomer) and found that the thickener solution had a salt resistance of 30 × 10^4^ mg/L and temperature resistance of 120 °C. Zhang et al. [30] developed a terpolymer with acrylamide, acrylic acid, and 4-isopropenylcarbamoyl-benzene sulfonic acid with high temperature and shear resistance using the aqueous solution polymerization method; the viscosity of the fracturing fluid was about 135 mPa s after 120 min at 150 °C and it had a shear rate of 170 s^−1^. Wei et al. [31] synthesized a W/O emulsion thickener by using acrylamide, acrylic acid, acrylamide-2-methylpropanesulfonic acid, and dimethyldiallyl ammonium chloride and realized the continuous mixing of slick water fracturing fluids; the drag reduction rate of the thickener was greater than 70% at a salinity of 2.5 × 10^4^ mg/L.

In recent years, many researchers in academic and industrial fields have improved the performance of polymers by introducing a small quantity of inorganic nanoparticles. Nano-silica is preferentially studied because of its nanometer size, high specific surface area, and large number of hydrogen bonds. Adding nanoparticles to a polymer solution or cross-linked fracturing fluid can improve the network structure of the fracturing fluid system, enhancing the strength, thermal and mechanical stability, and rheological properties of the fracturing fluid [32,33,34,35]. Fakoya et al. [32] found that the apparent viscosity and viscoelasticity of fracturing fluid can be significantly improved with an increase in the dosage of nano- silica. Liu et al. [33] investigated the influence of different nanoparticles (nano-silica, multiwall carbon nanotubes, graphene) on the mechanisms and performance of guar fracturing fluid and found that the interaction between nanomaterials and guar gum is mainly hydrogen bonds. The modified nanomaterials increase the dispersive force and hydrogen bonding between the nanomaterials and the thickener. Therefore, the addition of modified nanomaterials can enhance the apparent viscosity, temperature resistance, viscoelasticity, and mechanical properties of the fracturing fluid. However, different nanomaterials have different effects on the network structure of guar gum fracturing fluid. Nano-silica plays the role of a nuclear point and skeleton in the fracturing fluid, and its enhanced network structure effect is most obvious. Multiwalled carbon nanotubes are intertwined with guar gum macromolecular chains. Graphite powder intercalation enters the guar gum molecular chain, and the force is relatively weak. Nano-silica has the most outstanding enhancement effect on the performance of a fracturing fluid system, showing the best shear resistance, temperature stability, and viscoelasticity. Multiwalled carbon nanotube hybrid fracturing fluids are the second best option, while graphite powder hybrid fracturing fluids have the worst performance. Alharbi et al. [34] found that nanomaterials can reduce the viscosity loss of borate cross-linked fracturing fluid under high-pressure conditions. Xiang et al. [35] reported that a polymer/silica nanocomposite presents better performance in terms of temperature resistance, salt tolerance, shear resistance, and viscoelasticity than a pure polymer.

It is widely accepted that polymer/silica nanocomposites combine the advantages of fracturing fluids made from polymers and nanocomposites, and that nano-silica can enhance the stability of the suspension as an anti-sedimentation agent. However, the aforementioned studies do not mention the difference in performance between suspension and powder thickeners under the condition of having the same active polymer. Therefore, the main purpose of this study was to prepare a polymer suspension as the thickener for a fracturing fluid. Nano-silica and polyethylene glycol were used as the anti-sedimentation agent and solvent, respectively. A synthesized terpolymer with temperature resistance and salt resistance was used as the powder. The solubility, rheological behavior, sand-carrying performance, friction reduction characteristics, temperature and shear resistance ability, gel-breaking capacity, and microstructure of the suspension and powder thickener were systematically compared, so as to offer guidelines for field applications.

## 2. Results and Discussion

### 2.1. Characterization of Powder Thickener ACM

**Figure 1:**
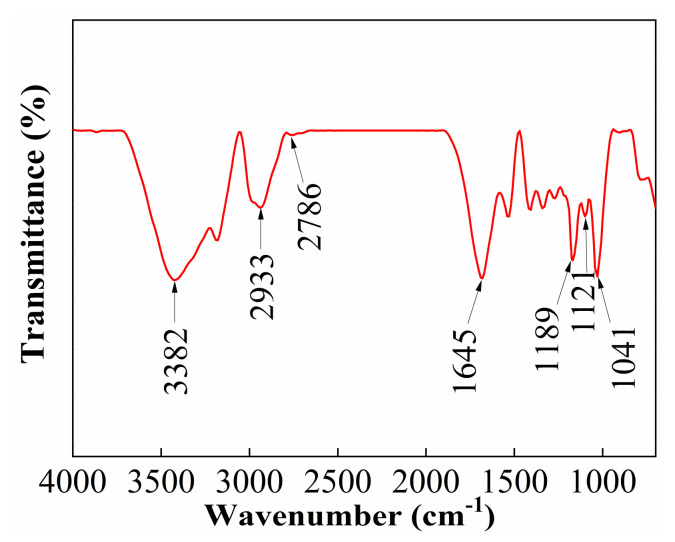
The FT-IR spectrum of ACM.

Figure 1 shows the FT-IR spectrum of ACM. The absorption peak at 3382 cm^−1^ is attributed to the stretching vibration of the N-H bond and the strong characteristic peak at 1645 cm^−1^ is related to the symmetric stretching vibrations of the C=O bond, further verifying the presence of AM in the polymer. The absorption peak at 1189 cm^−1^ is the stretching vibration of C=O and the peak at 1041 cm^−1^ is the asymmetric stretching vibration of the S=O bond, confirming the successful integration of AMPS into the polymer. The peaks at around 2933 cm^−1^ and 2786 cm^−1^ are attributed to the vibration absorption of the C-H bond in the ACMO the peak at 1121 cm^−1^ is assigned to the absorption band of the C-O bond in the ACMO, indicating that the ACMO was inserted successfully into the backbone of the polymer. The results of the FT-IR spectrum confirm that the polymer synthesized in this study is consistent with the designed molecule, which indicates that all monomers are involved in the reaction.

### 2.2. Stability Evaluation of Suspension SACM

**Figure 2:**
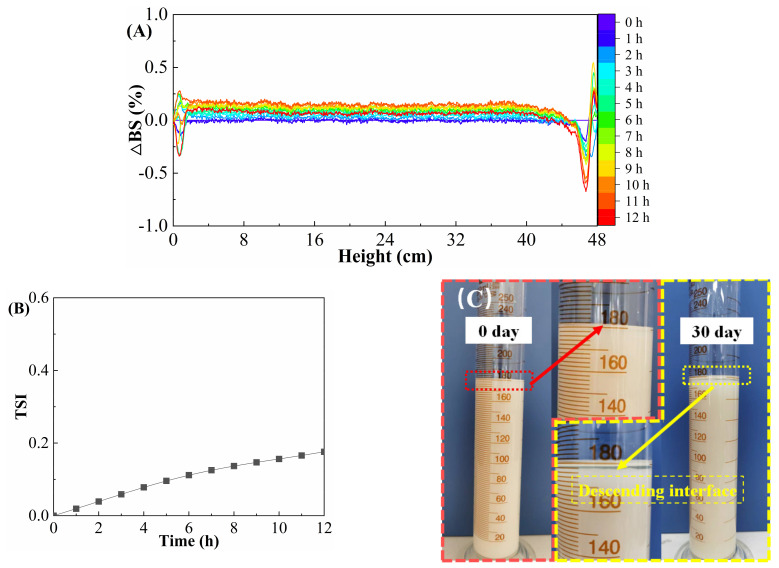
The stability evaluation of suspension SACM. (**A**) Back scattering spectra. (**B**) TSI values. (**C**) The status after standing for 30 days.

The ΔBS and TSI values of SACM were measured by a stability analyzer to evaluate its stability; the results are shown in Figure 2. As shown in Figure 2A, 0–4 mm and 44–48 mm represent the bottom and top of the sample tube, respectively. The blue curve and the red curve represent the data acquired at the start and the end of the scan, respectively. The ΔBS distribution curves of SACM show a horizontal trend, with absolute values of ΔBS ranging between 0 and 0.6%. The TSI curves (Figure 2B) of SACM present small variations with small values (range 0–0.17). The settling rate of SACM (Figure 2C) was less than 2.0% after standing for 30 days at 25 °C. The results of both the turbiscan lab measurement and settlement test indicate good stability of SACM. This is due to the small particle size and large specific surface area of the nano-silica, which possessed silanol groups on the surface of the silica particles. These silanol groups relied on interaction to form hydrogen bonds, forming a three-dimensional network structure that could enhance the viscosity of the suspension effectively, thus preventing the ACM powders from settling and improving the stability of the suspension [36]. The molecular chain of polyethylene glycol provided a spatial barrier shield, which effectively prevented the agglomeration of ACM powders. The adsorption of the agglomerated powders by the polyethylene glycol weakened the interconnections between the powders and played a certain role in stabilizing the powder.

### 2.3. Dissolution Rate

**Figure 3:**
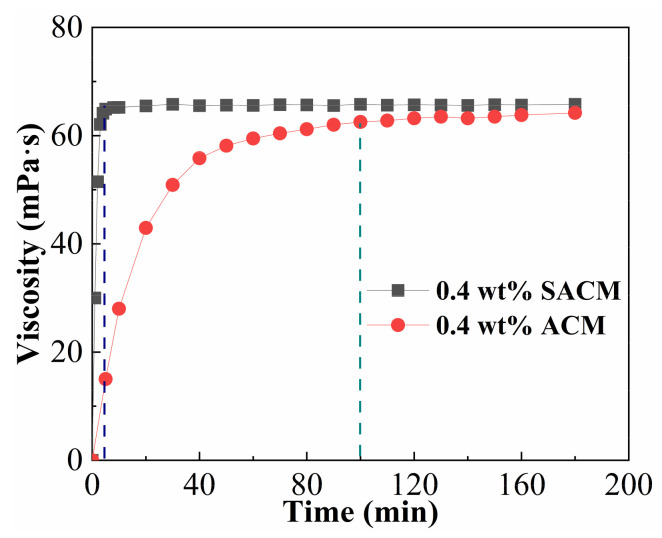
The viscosity–time plots of suspension and powder.

The instant dissolution of a thickener is an important parameter for determining whether it can be used for continuous fluid preparation in the fracturing process. When the thickener takes too long to dissolve, it can cause significant economic losses in field applications [37]. To certify the advantage of the instant dissolution of a thickener, we compared the viscosity–time plots of suspension and powder; the results are shown in Figure 3. The viscosity of the suspension diluent achieved its maximum value within 5 min, whereas the powder solution took 120 min to reach a stable value, indicating that the dissolution time of the suspension was much shorter than that of powder for the same thickener concentration. This is because the dissolution process of ACM powder is similar to that of common polymer powder, which swells first and then dissolves in water. Therefore, the dissolution of powders is usually very time consuming, causing certain difficulties for onsite operation. In [38], dispersed thickener powders in suspension were generally in the range of micrometers, so the dissolution did not need to undergo a swelling process; only a dilution of the concentrated solution was required, so the dissolution rate of the suspension was faster than that of the powder. It is worth noting that the maximum viscosity of the suspension diluent was slightly higher than that of the powder solution at the same thickener concentration. This result could be attributed to the physical adsorption of the thickener molecular chain on the surface of nano-silica, which made the system form a compact three-dimensional network structure and further improved the viscosity of the suspension diluent [39].

### 2.4. Rheological Behaviors

#### 2.4.1. Temperature Resistance

**Figure 4:**
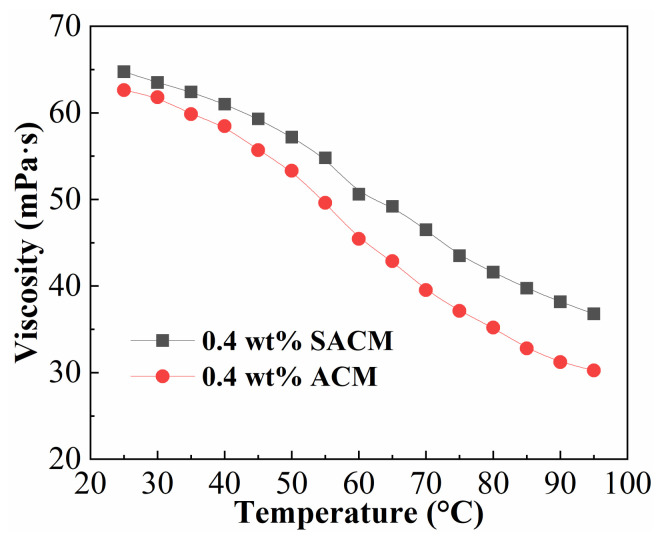
Effect of temperature on viscosity of suspension diluent and powder solution.

When fracturing fluids are injected into the target formation, the occurrence of heat exchange may result in unsatisfactory performance of the working fluid at high temperatures. Figure 4 shows the effect of temperature on viscosity of suspension diluent and powder solution. The viscosity of the two samples tended to decrease as the temperature increased from 25 °C to 95 °C. The intermolecular forces between polymer chains weakened with increasing temperature, leading to a decrease in viscosity. The morpholine group in ACMO introduced in the ASC could inhibit the curling of the molecular chain caused by the dehydration of the amide group and the sulfonic acid group; thus, the thickener solution possessed good temperature resistance at 95 °C. However, it should be noted that the suspension diluent exhibited higher viscosity than the powder solution because the large steric hindrance of the nano-silica restricted the movement of the thickener chains and enhanced chain rigidity. In addition, the presence of strong bonds between Si-O and C-Si in the suspension diluent weakened the degradation of the thickener chains compared with powder solution, which means that more energy needed to be consumed in the dissociation between nano-silica and thickener [40]. This synergistic effect gives the suspension diluent outstanding temperature resistance.

#### 2.4.2. Salt Resistance

**Figure 5:**
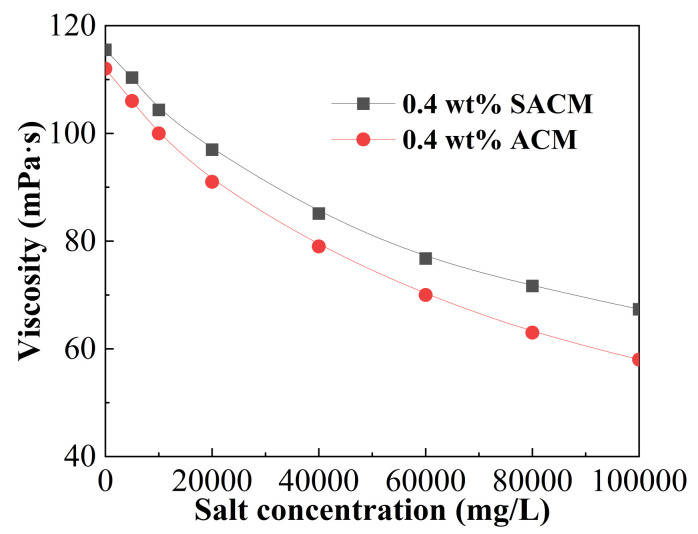
Effect of salinity on viscosity of suspension diluent and powder solution.

Flowback water is commonly recycled for the preparation of fracturing fluids in some oil fields in western China. However, the relatively high salinity of flowback water has a negative impact on performance. Figure 5 shows the effect of salinity on viscosity of suspension diluent and powder solution. The viscosity of powder solution gradually decreased with the increase in salt concentration; a similar variation for the suspension diluent in terms of salinity was observed over the entire test range. The presence of salts reinforced the solution polarity and weakened electrostatic repulsion of the thickener solution, which caused the curl of thickener molecular chains and decreased the hydrodynamic volume and viscosity of the thickener solution [41]. However, due to the electronegativity shielding effect of the sulfonic acid group in AMPS and rigid morpholine group in ACMO, which played a significant role in supporting the backbone, the molecular structure of the thickener was stable and the volume shrinkage of the molecular chain was limited, so that the thickener molecules still maintained high viscosity under a high salt concentration [29].The viscosity of the suspension diluent was higher than that of the powder solution under the same experimental conditions. This phenomenon could be attributed to the addition of nano-silica. As inorganic nanoparticles, nano-silica was insensitive to the metal ions (such as Na^+^ and Ca^2+^) and provided steric hindrance to the inhibitory effect of electric double layers of the thickener hydration shell, leading to a bigger hydrodynamic volume and higher viscosity [42]. Therefore, the suspension diluent showed better salt resistance than the powder solution.

#### 2.4.3. Shear Resistance

**Figure 6:**
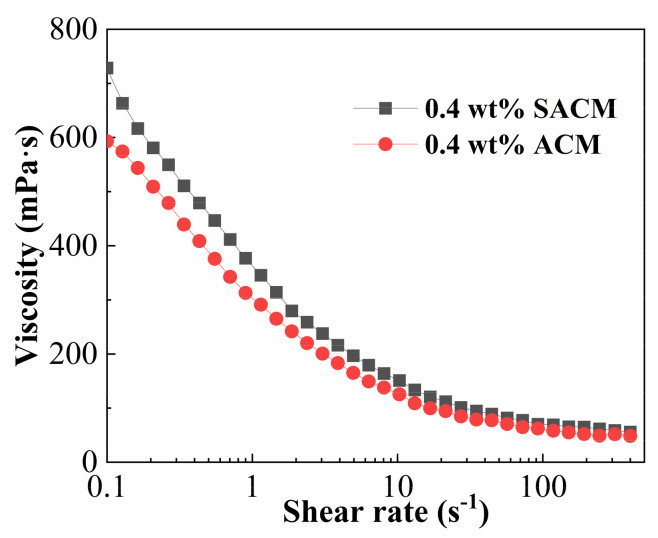
Effect of shear rate on viscosity of suspension diluent and powder solution.

During hydraulic fracturing, fracturing fluids are affected by mechanical degradation when they move from the ground pump units to the target reservoir through pipelines; viscosity is commonly applied to assess the shear resistance of the fracturing fluids under variable shear conditions. The viscosity–shear rate curves are shown in Figure 6. Two samples exhibited a shear thinning characteristic with the increasing shear rate. The interactions between molecular chains of the thickener were disrupted with an increase in shear rate, so the viscosity decreased significantly. It is worth noting that the viscosity of the suspension diluent decreased more slowly than the viscosity of powder solution for increasing shear rates. At 400 s^−1^, the retention rates of the viscosity of suspension diluent and powder solution were 42.1% and 38.6%, respectively. This was because the presence of nano-silica enhanced the stability of the thickener solution; nanoparticles provided many cross-linking points, resulting in the complex structure of solutions, and the rigidity of nanoparticles improved the mechanical properties of thickener chains. The interaction between the nano-silica and the thickener molecular improved the strength of the macromolecular network through the formation of cross-linked structures [43]. As a result, the suspension diluent showed a higher viscosity than the powder solution under continuous shear action. However, the difference in viscosity was much smaller at a high shear rate, which means that the shear resistance should be further increased.

#### 2.4.4. Viscoelasticity

Viscoelasticity is a vital indicator for evaluating the sand-carrying property of a fracturing fluid, which is an important parameter for characterizing the performance of fracturing fluids. Therefore, it is necessary to measure the loss modulus (G”) and storage modulus (G’) of a thickener solution. The inverse of the intersection point of storage modulus and loss modulus in the frequency scanning curve represents the relaxation time of the thickener solution. The lower the intersection value, the stronger the proppant-carrying capacity [44]. To a certain extent, the elasticity of the fracturing fluid reflects the density of the network structure in the solution. The stronger the spatial network structure, the greater the storage modulus, indicating more extensive network structures in fracturing fluids.

**Figure 7:**
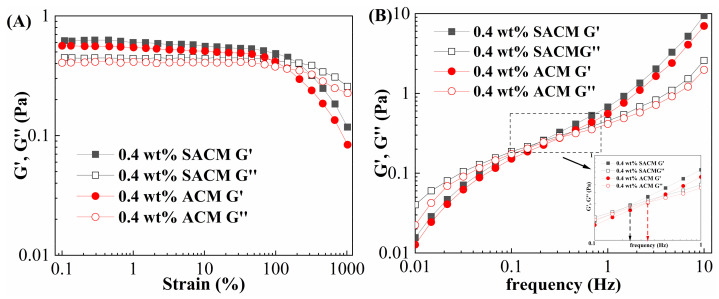
The viscoelasticity of suspension diluent and powder solution. (**A**) Strain scanning curve. (**B**) Frequency scanning curve.

Figure 7 present the viscoelasticity testing results for suspension diluent and powder solution. As shown in Figure 7A, the strain sweep was designed to determine the linear viscoelastic region of the thickener solution. The range of the linear viscoelastic zone of the suspension diluent was larger than that of powder solution. The storage modulus was higher than the loss modulus in the linear viscoelastic zone, indicating a predominance of elastic behavior. Taking into account the results of strain scanning, the strain was set as 10% for frequency scanning tests. Figure 7B shows the storage modulus and loss modulus as a function of frequency. In the course of testing, the suspension diluent presented higher values of storage modulus and loss modulus and a lower cross point value. This was probably due to the strong interactions between nano-silica and the hosting thickener matrix. The well-dispersed nano-silica adsorbed onto the polymeric matrix and cross-linked with more molecular chains, so there was an increase in the network junction density [45]. The network structure was strengthened due to more effective connection points, which facilitated the viscoelastic behavior of the thickener. Both viscosity and viscoelasticity measurements indicated that the nano-silica contributed to a stronger polymer network, which was more effective in carrying proppants.

### 2.5. Sand-Carrying Property

**Figure 8:**
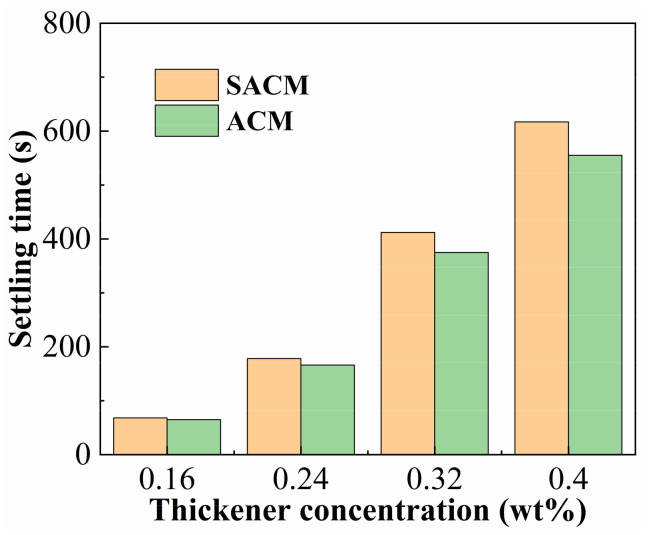
Sand-setting time of suspension diluent and powder solution.

It is necessary to measure the sand-carrying property of suspension diluent and powder solution. Thickener solutions of different concentrations were chosen to test the average time it took for five particles of ceramsite to fall in a volume of 250 mL; the results are shown in Figure 8. When the thickener concentration was lower than 0.24 wt%, the settling time of the proppant in both kinds of thickener solutions was basically the same. When the concentration of powder reached 0.40 wt%, the proppant achieved a settling time of 556 s under static conditions, indicating that the powder possessed a good sand-carrying property. During the actual fracturing process, the thickener solution was flowing at high speed, so the sand-carrying property was greater. The settling time of the 0.40 wt% suspension solution increased to 617 s; the settling time of the suspension solution was apparently higher than that of the powder solution when the thickener concentration was larger than 0.40 wt%. This indicates that the sand-carrying performance of the thickener solution was improved after the addition of nanomaterials [33].

### 2.6. Drag Reduction Performance

**Figure 9:**
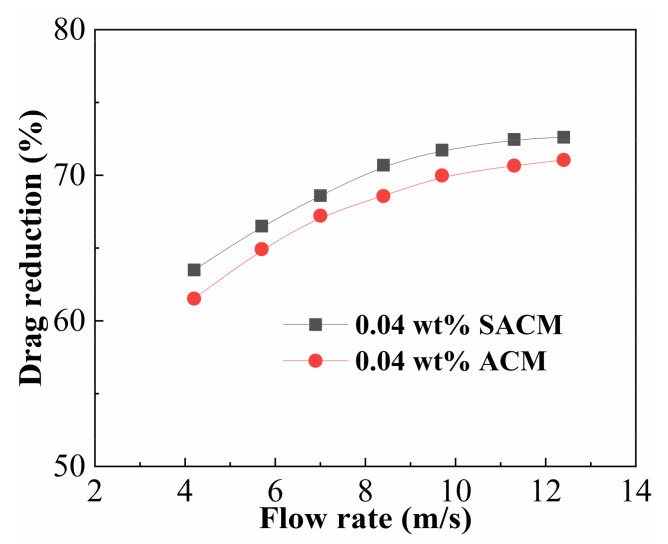
Drag reduction curves for suspension diluent and powder solution.

Figure 9 compares the experimental results of drag reduction performance for suspension diluent and powder solution at a thickener concentration of 0.04 wt%. The drag reduction rate increased when the flow rate increased from 2.4 m/s to 10.2 m/s; the maximum drag reduction rate was 72.6% and 70.9% for suspension diluent and powder solution, respectively. When the thickener solution started to flow, the conformation of thickener molecules changed from the coil to extension under external flow, resulting in a strong inhibition of the formation and further development of vortices, so the drag reduction rate increased gradually. As the flow rate increased, the molecular chains reached full extension and the confinement of turbulence vortices became more effective, corresponding to the maximum drag reduction rate [35]. The drag reduction rates of suspension and powder had a similar variation trend, but the drag reduction rates of the suspension were higher than those of the powder at the same flow rate, and a wider range of high drag reduction was obtained by the suspension. This result could be attributed to the reinforced interactions between the nano-silica and the thickener matrix, When the nanosilica was introduced, it provided more bridge nodes and enhanced the strength of the spatial network. The well-dispersed nano-silica in the thickener matrix could reduce the degradation of thickener molecules by improving molecular rigidity, enhancing the resistance to the shear action and hindrance to vortices, thus showing better drag reduction performance in the turbulent state [46].

### 2.7. Temperature and Shear Resistance Performance

**Figure 10:**
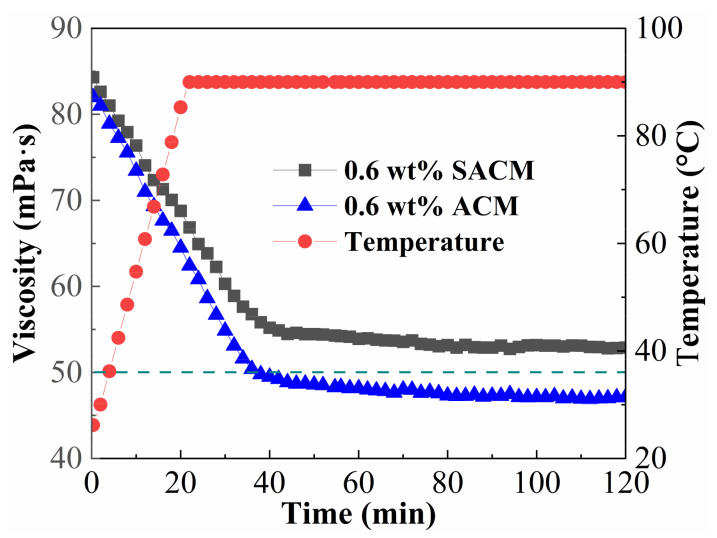
Temperature and shear resistance performance of suspension diluent and powder solution.

The ability of a fracturing fluid to sustain high viscosity under conditions of high temperature and mechanical shearing is one of the keys to successful fracturing. Figure 10 shows temperature and shear resistance performance of suspension diluent and powder solution at a temperature of 90 °C and a shear rate of 100 s^−1^. The viscosity of the two kinds of solutions decreased with the increase in temperature. After the temperature rose to the predetermined value, the viscosity decreased slowly and then maintained a stable trend during continuous shearing. The suspension diluent and powder solution displayed a stable viscosity of 52.9 and 47.1 mPa·s and a viscosity retention rate of 62.7% and 58.1%, respectively. The results clearly show that the well-dispersed nano-silica was helpful in terms of improving the temperature and shear resistance performance of the thickener solution. The nano-silica was dispersed in the thickener solution, which reduced the free volume of the system and limited the movements of thickener chains. In addition, nano-silica acted as a physical cross-linking agent stabilizing the spatial network of the thickener solution, while the rigidity of nano-silica enhanced the mechanical properties of thickener molecule chains [47,48].

### 2.8. Gel Breaking

Once the fracturing operation is complete, the viscosity of the fracturing fluid must be reduced quickly to allow for easy flowback. Therefore, the gel-breaking performance and residue content of suspension diluent and powder solution were tested; the results are shown in Table 1. The gel-breaking times of both suspension diluent and powder solution were 4 h, and the viscosities of gel-breaking liquids were 2.5 mPa s and 2.2 mPa·s, respectively. Compared with suspension diluent, the powder solution was broken more easily with less residual content and lower viscosity, which allowed better fracture conductivity. The residual content of suspension diluent and powder solution was 67.2 and 53.6 mg/L, respectively; these values are below the limit imposed on the petroleum industry in China (200 mg/L). The suspension diluent had low residual content and low damage to reservoirs, so it possesses good prospects for application.

### 2.9. Microstructure Analysis

**Figure 11:**
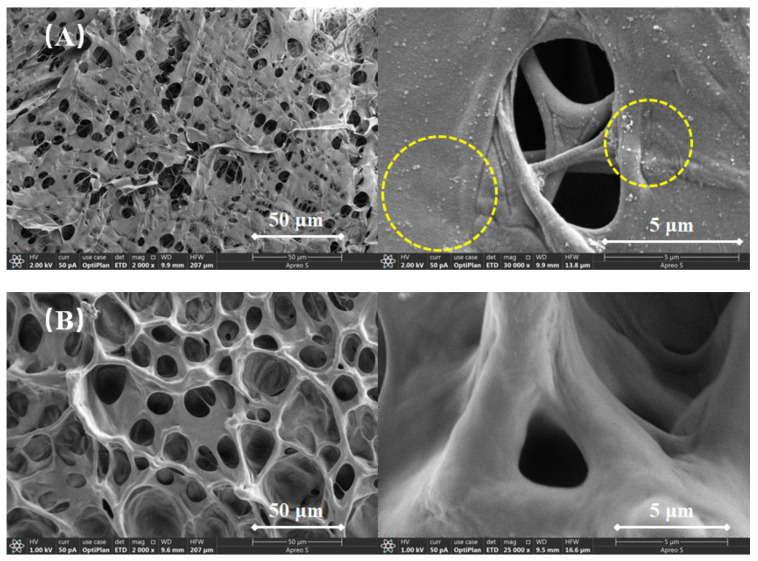
SEM images of (**A**) suspension diluent and (**B**) powder solution.

The morphology of the thickener solution with a thickener concentration of 0.4 wt% was analyzed in depth by SEM. Figure 11A,B shows the SEM images of suspension diluent and powder solution. Although these two fluids exhibited similar network structures, the network structure became denser with the introduction of nano-silica. Nano-silica particles (yellow dotted line) acted as connection points between the thickener chains, resulting in a denser skeleton. In addition, the dispersed nano-silica remained relatively uniformly distributed in the fracturing fluid, without visible agglomeration.

**Figure 12:**
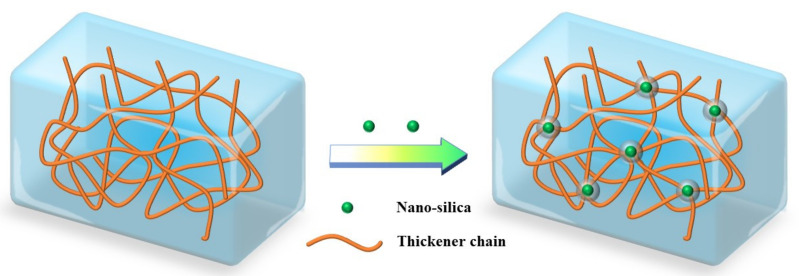
Schematic diagram of the interactions between the thickener matrix and the nano-silica.

Figure 12 helps to visualize the reasons for the behavior of the suspension diluent. Firstly, the mobility of thickener chains surrounding the nano-silica in solution was inhibited by the steric hindrance of the nano-silica, leading to an increased stability of the network. Secondly, nano-silica particles acted as crosslinkers by attaching several molecule chains. At the same time, each thickener chain might be absorbed by a different nano-silica particle. This bridging action resulted in the formation of the complex macromolecule network, which improved the structure strength. In addition, the presence of hydrogen bonds between the thickener matrix and the nano-silica restricted the local movement of thickener chains, leading to a stronger spatial network [49]. As a result, these synergistic effects greatly enhanced the performance of the suspension diluent.

## 3. Conclusions

In this paper, a powder thickener (ACM) was synthesized using AM, ACMO, and AMPS via free radical copolymerization. A novel suspension of a thickener as a fracturing fluid (SACM) was prepared using ACM, nano-silica, and polyethylene glycol under the condition of high-speed stirring. The solubility, rheological behavior, sand-carrying performance, drag reduction characteristics, temperature and shear resistance ability, gel-breaking capacity, and microstructure of a suspension and powder thickener were systematically compared. The suspension was characterized by a shorter dissolution time (5 min) than the powder thickener (120 min), allowing it to meet the requirements of large-scale mixing and increased economic benefits. The suspension exhibited better performance in temperature resistance, salt resistance, shear resistance, viscoelasticity, sand carrying, and drag reduction than powder thickener under the same experimental conditions. SEM showed that the nano-silica particles acted as connection points between the thickener chains, which enhanced the rigidity of the thickener molecular chains and strengthened the network structure of the thickener solution, leading to a stronger spatial network. As a result, these synergistic effects greatly enhanced the performance of the suspension. Although the powder thickener was broken more easily with less residual content and lower viscosity, the suspension diluent had low residual content and low damage to reservoirs, below the limit imposed on the petroleum industry in China. Due to the above excellent properties, the suspension could further improve fracturing effects and has the potential to be used in fracturing fluids for reservoir stimulation.

## 4. Materials and Methods

### 4.1. Materials

Acrylamide (AM, AR, 99%), 2-acrylamide-2-methylpropanesulfonic acid (AMPS, AR, 98%), acryloyl morpholine (ACMO, AR, 98%), sodium hydroxide (NaOH, AR, 98%), sodium chloride (NaCl, AR, 99.5%), calcium chloride (CaCl_2_, AR, 98%), 2,2-Azobis (2-methylpropionamide) dihydrochloride (V50, AR, 98%), polyethylene glycol 400 (PEG 400, AR, 99%), ammonium persulfate (APS, AR, 98%), and potassium bromide (KBr, AR, 99%) were provided by Aladdin Reagent Co., Ltd. (Shanghai, China). Nano-silica particles (industrial grade, 99%) with an average particle diameter of 20 nm were purchased from Newthink New Materials Co., Ltd. (Qingdao, China). Simulated brine prepared with sodium chloride and calcium chloride at a mass ratio of 10:1 was synthesized in the lab, intended to simulate water with a salinity of 100,000 mg/L in western China oilfields. Deionized (DI) water was obtained from a water purification system. Proppant ceramsite with an apparent density of 2.45 g/cm^3^ and a size of 40/70 mesh was supplied by Jingang New Materials Co., Ltd. (Zouping, China). All chemicals and reagents were utilized without further purification.

### 4.2. Synthesis of Thickener

#### 4.2.1. Synthesis of Powder Thickener

**Figure 13:**
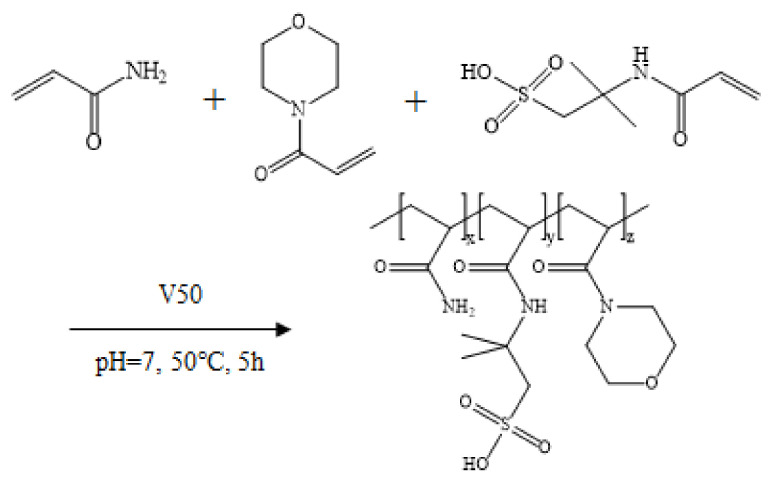
The synthesis process of ACM.

The synthetic reaction was conducted in a three-necked flask equipped with a magnetic stirrer. Certain proportions of AM (91.6 g, 1.289 mol), AMPS (20.8 g, 0.100 mol), and ACMO (7.8 g, 0.055 mol) were added to deionized water under an inert nitrogen atmosphere for 30 min; the total monomer concentration was maintained at 30 wt%, and the pH value was adjusted to 7.0 by using sodium hydroxide solution. V50 solution serving as the initiator was added using a syringe; the dosage of V50 was 0.1 wt% of the total mass of the monomers. The solution was stored at 50 °C for 5 h. The obtained product was cut into small pieces and purified by using ethanol precipitation three times. Finally, the powder thickener was acquired after vacuum drying and granulation; the powder thickener had a size of 100/120 mesh and was named ACM. The synthesis process of ACM is presented in Figure 13.

#### 4.2.2. Characterization of Powder Thickener by Infrared Spectroscopy

The Fourier transform infrared (FT-IR) spectrum of the ACM was obtained by using an IRTracer-100 infrared spectrometer (SHIMADAZU, Japan) in the 4000–700 cm^−1^ range with a resolution of 4 cm^−1^ at room temperature. The FT-IR sample was prepared by grinding and pressing the mixture containing the ACM powders and KBr powders at a mass ratio of 1:75.

#### 4.2.3. Preparation of Suspension

The suspension was prepared according to reported methods [24]. The process was as follows: Appropriate amounts of PEG-400 and nano-silica were poured into a beaker, and the alcohol-soluble suspension was obtained by stirring at a rate of 1500 rpm for 30 min. Then, ACM powders were added to the alcohol-soluble suspension during stirring. The stirring lasted for 240 min at a rate of 700 rpm to generate the homogeneous suspension, which was named SACM, with an active content of 40 wt%.

### 4.3. Stability Tests

#### 4.3.1. Turbiscan Lab Measurement

The rate of the backscattered light (ΔBS) and Turbiscan stability index (TSI) values of the SACM were measured by a Turbiscan Lab stability analyzer (Formulaction Company, Toulouse, France) to analyze its static stability for 12 h. The scanning height was 48 mm and the test temperature was 25 °C. We set a near-infrared light source (λ = 850 nm) and scanned the sample along the axial height every 40 μm; the ACM was scanned 12 times in 12 h.

#### 4.3.2. Sedimentation Rate

A certain amount of SACM was transferred to cylinder and allowed to settle naturally for 30 days. The descending distance of the interface at different times at 25 °C was recorded. The ratio of descending distance of the interface to initial liquid level height was defined as the sedimentation rate.

### 4.4. Dissolution Rate of the Thickener

For the suspension, the dissolution was based on the following steps: In a 800 mL beaker, 4.0 g of SACM was quickly injected into 396 g of 100,000 mg/L simulated brine to obtain a suspension diluent with a thickener concentration of 0.4 wt% under gentle stirring (300 rpm). For the powder thickener, a mechanical stirrer was adjusted to 300 rpm, the designed dosages of ACM were subsequently added to the 100,000 mg/L simulated brine, and then rotational speed was regulated to a low gear at 100 rpm to obtain 0.4 wt% thickener solution. Every 1 or 5 min, the suspension or powder solution was taken out for viscosity measurement by using a HAKKE MARS40 rheometer (HAAKE, Karlsruhe, Germany) with Cup Z43 cylinder plate (diameter = 43 mm) and CC41 rotor (diameter = 41 mm) at a shear rate of 170 s^−1^ and 25 °C.

### 4.5. Rheological Characteristic Test

#### 4.5.1. Thickener Solution Property Measurements

The HAKKE MARS40 rheometer with Cup Z43 cylinder plate and CC41 rotor was used to measure the solution property of suspension and powder thickener. The dedicated amounts of suspensions and powders were dissolved in simulated brine with a salinity of 100,000 mg/L to acquire thickener solutions. Temperature resistance measurements of suspension diluent and powder solutions were carried out over a temperature range of 25–95 °C at a concentration of 0.4 wt% and a shear rate of 170 s^−1^. Salt resistance tests were recorded in different salinities of simulated brine with a thickener concentration of 0.4 wt% at 25 °C and a shear rate of 170 s^−1^. The shear resistance analyses were carried out by a continuous shear in the range from 0.1 to 400 s^−1^ at 25 °C for 25 min. The viscosity retention rate was calculated by the viscosity at 400 s^−1^ divided by the viscosity at 10 s^−1^.

#### 4.5.2. Viscoelasticity Test

The suspension diluent and powder solution were prepared with 100,000 mg/L simulated brine. The HAAKE MARS40 rheometer was used to measure the viscoelasticity of the suspension diluent and powder solution. The cone plate geometry systems with cone PP35/Ti (diameter was 35 mm, gal was 1 mm) was selected for the measurement. The viscoelastic properties were determined under the oscillatory shearing conditions at 25 °C.

When testing the viscoelasticity of a sample, the experiment must be carried out in the linear viscoelastic region of the sample. If the selected strains are not in the linear viscoelastic region, then large amplitudes may damage the structure of the sample, resulting in shear dilution, which may lead to unexplained deviations in the sample data tested with different instruments and experimental conditions. Therefore, the strain amplitude ranged from 0.1 to 1000% at an oscillation frequency of 1 Hz to determine the linear viscoelastic zone. The strain values of thickener solution in the linear viscoelastic region were used to conduct the frequency scanning of the solution to determine the viscoelastic strength, and the range of frequency was 0.01–10 Hz.

### 4.6. Sand-Carrying Property

We prepared 0.4 wt% suspension diluent and powder solution with 100,000 mg/L simulated brine and placed them in a 250 mL cylinder. We then placed five grains of ceramsite into the measuring cylinder, recorded the time it took for the ceramsite to drop from 250 to 0 mL, and then calculated the average time it took for the ceramsite to settle at 25 °C [50].

### 4.7. Drag Reduction Measurement

The suspension diluent and powder solution were prepared with simulated brine of 100,000 mg/L salinity, and the drag reduction rate of thickener solutions was measured using a self-made loop drag test system [51]. In this experiment, a pipe with an inner diameter of 8 mm and length of 3.3 m was chosen. Next, the pressure drops of water flowing without a reagent were measured at 25 °C. Then, suspension diluent or powder solution was prepared. The pressure drops of suspension diluent or powder solution were obtained after going through the pipeline at the same flow rate, and the drag reduction rate was calculated according to Equation (1).

(1) $$DR\%=\frac{\Delta P_{1}-\Delta P_{2}}{\Delta P_{1}}\times 100\%$$

Here, *DR* is drag reduction rate (%), Δ*P*_1_ is the pressure drop generated in simulated brine (KPa), and Δ*P*_2_ is the pressure drop measured in suspension diluent or powder solution (KPa).

### 4.8. Temperature and Shear Resistance Performance

The suspension diluent and powder solution with a thickener concentration of 0.6 wt% were prepared with 100,000 mg/L simulated brine. First, the tested liquid was heated from 25 to 90 °C at a heating rate of 3 °C/min under a shear rate of 100 s^−1^ by using the HAKKE MARS40 rheometer. Then, the temperature and shear resistance performance of the liquid was measured at a temperature of 90 °C and a shear rate of 100 s^−1^ for 100 min. The rheometer utilized a high-pressure sealed concentric cylinder and rotor (PZ 38b), which required a sample volume of 32 mL.

### 4.9. Microstructure Analysis

The microstructure of the suspension diluent and powder solution was observed by scanning electron microscope (SEM, Quanta 450, FEI, Portland, OR, USA). All samples were frozen in liquid nitrogen at −50 °C, and the morphologies of the frozen samples were observed with the SEM operating at an accelerating voltage of 2.0 kV.

### 4.10. Gel-Breaking Test

The suspension diluent and powder solution with thickener concentration of 0.6 wt% prepared with 100,000 mg/L simulated brine were placed in respective beakers, to which 0.06 wt% ammonium persulfate as the gel breaker was added. The thickener solutions including breakers were stirred uniformly and then put into a constant-temperature water bath at 90 °C for 4 h. A HAKKE MARS40 rheometer was also used to measure the viscosity of the broken fluids under a shear rate of 170 s^−1^ at 25 °C. The residue was obtained by centrifugation and filtration of broken fluids; then, after filtering, the filter paper was placed in an oven to dry at 100 °C for 8 h. The amount of residues was calculated by the difference in the weight of the filter paper before and after the experimental treatment.

## Figures and Tables

**Table 1:** Gel-breaking test of suspension diluent and powder solution.

No	Type of Thickener	Apparent Viscosity of Gel-Breaking Solution (mPa·s)				Residual Content (mg/L)
		1 h	2 h	4 h	6 h	
1	suspension	32.6	15.8	3.4	2.5	67.2
2	powder	24.3	13.8	3.0	2.2	53.6

## Data Availability

The data presented in this study are available upon request from the corresponding author.
